# Evaluating the Impact of Pharmacotherapy in Augmenting Quit Rates Among Hispanic Adults in an App-Delivered Smoking Cessation Intervention: Secondary Analysis of a Randomized Controlled Trial

**DOI:** 10.2196/69311

**Published:** 2025-01-31

**Authors:** Margarita Santiago-Torres, Kristin E Mull, Brianna M Sullivan, Ana Paula Cupertino, Ramzi G Salloum, Matthew Triplette, Michael J Zvolensky, Jonathan B Bricker

**Affiliations:** 1 Division of Public Health Sciences Fred Hutchinson Cancer Center Seattle, WA United States; 2 Department of Public Health Sciences University of Rochester Medical Center Rochester, NY United States; 3 Department of Health Outcomes and Biomedical Informatics, College of Medicine University of Florida Gainesville, FL United States; 4 Division of Pulmonary, Critical Care and Sleep Medicine, Department of Medicine University of Washington Seattle, WA United States; 5 Clinical Research Division Fred Hutchinson Cancer Center Seattle, WA United States; 6 Department of Psychology University of Houston Houston, TX United States; 7 MD Anderson Cancer Center University of Texas Houston, TX United States; 8 Department of Psychology University of Washington Seattle, WA United States

**Keywords:** acceptance and commitment therapy, Hispanic or Latino, iCanQuit, QuitGuide, smartphone apps, smoking cessation, mobile phone

## Abstract

**Background:**

Hispanic adults receive less advice to quit smoking and use fewer evidence-based smoking cessation treatments compared to their non-Hispanic counterparts. Digital smoking cessation interventions, such as those delivered via smartphone apps, provide a feasible and within-reach treatment option for Hispanic adults who smoke and want to quit smoking. While the combination of pharmacotherapy and behavioral interventions are considered best practices for smoking cessation, its efficacy among Hispanic adults, especially alongside smartphone app–based interventions, is uncertain.

**Objective:**

This secondary analysis used data from a randomized controlled trial that compared the efficacy of 2 smoking cessation apps, iCanQuit (based on acceptance and commitment therapy) and QuitGuide (following US clinical practice guidelines), to explore the association between pharmacotherapy use and smoking cessation outcomes among the subsample of 173 Hispanic participants who reported on pharmacotherapy use. Given the randomized design, we first tested the potential interaction of pharmacotherapy use and intervention arm on 12-month cigarette smoking abstinence. We then examined whether the use of any pharmacotherapy (ie, nicotine replacement therapy [NRT], varenicline, or bupropion) and NRT alone augmented each app-based intervention efficacy.

**Methods:**

Participants reported using pharmacotherapy on their own during the 3-month follow-up and cigarette smoking abstinence at the 12-month follow-up via web-based surveys. These data were used (1) to test the interaction effect of using pharmacotherapy to aid smoking cessation and intervention arm (iCanQuit vs QuitGuide) on smoking cessation at 12 months and (2) to test whether the use of pharmacotherapy to aid smoking cessation augmented the efficacy of each intervention arm to help participants successfully quit smoking.

**Results:**

The subsample of Hispanic participants was recruited from 30 US states. They were on average 34.5 (SD 9.3) years of age, 50.9% (88/173) were female, and 56.1% (97/173) reported smoking at least 10 cigarettes daily. Approximately 22% (38/173) of participants reported using pharmacotherapy to aid smoking cessation at the 3-month follow-up, including NRT, varenicline, or bupropion, with no difference between intervention arms. There was an interaction between pharmacotherapy use and intervention arm that marginally influenced 12-month quit rates at 12 months (*P* for interaction=.053). In the iCanQuit arm, 12-month missing-as-smoking quit rates were 43.8% (7/16) for pharmacotherapy users versus 28.8% (19/16) for nonusers (odds ratio 2.21, 95% CI 0.66-7.48; *P*=.20). In the QuitGuide arm, quit rates were 9.1% (2/22) for pharmacotherapy users versus 21.7% (15/69) for nonusers (odds ratio 0.36, 95% CI 0.07-1.72; *P*=.20). Results were similar for the use of NRT only.

**Conclusions:**

Combining pharmacotherapy to aid smoking cessation with a smartphone app–based behavioral intervention that teaches acceptance of cravings to smoke (iCanQuit) shows promise in improving quit rates among Hispanic adults. However, this combined approach was not effective with the US clinical guideline–based app (QuitGuide).

**Trial Registration:**

ClinicalTrials.gov NCT02724462; https://clinicaltrials.gov/study/NCT02724462

**International Registered Report Identifier (IRRID):**

RR2-10.1001/jamainternmed.2020.4055

## Introduction

### Background

In the United States, 1 in 5 adults are Hispanic or Latino (referred to hereafter as Hispanic). Hispanic adults in the United States constitute the largest ethnic minority group [1,2]. In fact, it is estimated that by 2026, the Hispanic population will reach 111 million [3]. Presently, there are approximately 4 million Hispanic adults who smoke cigarettes [4]. Even though smoking rates are lower among Hispanic adults (7.7% vs 11.7%) when compared to the general population of adults in the United States who smoke [5], the prevalence of smoking varies across different Hispanic nationalities [6-8]. For instance, people from Puerto Rico and Cuba have higher than average smoking rates at 25.5% and 19.8%, respectively. Hispanic adults also face higher smoking-attributable morbidity and mortality compared to their non-Hispanic White counterparts [9-11]. Despite these health inequities in smoking and the rapid population growth of the Hispanic population in the United States, research on smoking cessation interventions for this group is sparse [12], with only 2 full-scale randomized controlled trials (RCTs) with 6-month follow-up or longer conducted to date [13,14].

While evidence-based smoking cessation treatments are generally within reach of the US general adult population [15,16], Hispanic adults often lack access to these treatments [17]. Reasons for these accessibility barriers include health providers being 50% less likely to offer quit-smoking advice to Hispanic adults compared to their non-Hispanic counterparts, which greatly contributes to significant inequities in smoking cessation efforts in this group [4,17]. Moreover, many Hispanic adults attempt to quit cigarette smoking cold turkey, which typically results in a very low likelihood of success of 2% to 5% [18,19]. Addressing these challenges requires research to develop culturally inclusive behavioral interventions that are within reach and tailored to the unique needs of Hispanic adults who smoke and want to quit smoking.

Free and remotely delivered digital interventions may improve access to evidence-based smoking cessation programs and thereby help improve smoking cessation outcomes [20]. Only 1 RCT, with a follow-up of 6 months or longer, has examined a digital smoking cessation intervention for Hispanic adults [21]. This study randomly assigned 457 English and Spanish-speaking Hispanic adults to receive either a culturally tailored mobile health smoking cessation intervention (Decídetexto) or standard of care, including written materials from the American Cancer Society and the National Cancer Institute (NCI), along with access to state-funded tobacco quitlines. Nicotine replacement therapies (NRTs; patches and gum) were offered to both arms at no cost. Results at 6 months showed higher (but nonsignificant) biochemically verified quit rates (Decídetexto: 33/229, 14.4% vs control group: 21/228, 9.2%; *P*=.09) and greater NRT use in the intervention group compared to standard of care (at least 1 day of NRT: 174/192, 90.6% vs 139/198, 70.2%; *P*=.01). However, it remains unclear if the use of pharmacotherapy to aid smoking cessation contributed to these quit rates, as both groups had access to free medication.

Digital interventions, like smartphone apps, offer an alternative approach to SMS text messaging or website-based interventions for smoking cessation [22], with over 500 smoking cessation apps currently available (R Nelson, personal communication, 2020). Although 91% of Hispanic adults in the United States own smartphones [23], to our knowledge, only 1 study has explored the potential efficacy of an app-based intervention for helping Hispanic adults quit smoking [24]. This study was a secondary analysis of a Hispanic adult subsample (n=210, 8.7% of the total sample) from a full-scale RCT comparing the iCanQuit app (Moby, Inc; based on acceptance and commitment therapy [ACT]) with the NCI’s QuitGuide app (National Cancer Institute; based on US clinical practice guidelines [USCPGs]) for smoking cessation [24,25]. Results showed significantly higher 12-month quit rates in iCanQuit (28/82, 34.1%) versus QuitGuide (19/94, 20.2%; odds ratio [OR] 2.20, 95% CI 1.10-4.41; *P*=.02). This finding is significant, given the challenges faced by Hispanic adults in accessing evidence-based smoking cessation treatments, such as lack of medical insurance, ethnic discrimination, and cost [26,27].

Although app-based smoking cessation interventions hold promise, USCPGs emphasize that combining behavioral interventions with pharmacotherapy to aid smoking cessation is more efficacious than either approach alone [28,29]. In the iCanQuit parent RCT, pharmacotherapy use was an exclusion criterion, but participants could opt to use it on their own after randomization [25]. In fact, both the iCanQuit and QuitGuide app–based interventions educate users on Food and Drug Administration (FDA)–approved pharmacotherapy to aid cessation [25]. In the full sample of the iCanQuit parent RCT, participants in the iCanQuit arm who used pharmacotherapy to aid smoking cessation on their own had significantly higher quit rates at 12 months compared to nonusers (users: 70/176, 39.8% vs nonusers: 192/691, 27.8%; *P*=.002) [30]. Whether the use of pharmacotherapy to aid cessation improved the quit rates of Hispanic participants, specifically, remains unknown.

Hispanic adults in the United States have the lowest rate of pharmacotherapy use to aid smoking cessation compared to other racial or ethnic groups [4,17,31]. For example, the rate of pharmacotherapy use is approximately 25.2% among non-Hispanic Black adults and 23.6% among non-Hispanic White adults but only 16.6% among Hispanic adults. In fact, reports from population-based studies of NRT use during quit attempts showed that an overwhelming 91% of Hispanic adults had never used NRT to aid their quit attempts [19]. There are various reasons why Hispanic adults may be more reluctant to use pharmacotherapy to aid their quit attempts. For instance, financial barriers and being medically uninsured (24% of US Hispanic adults) may play an important role [32,33]. Other reasons may include having a mistrust of the medical system [34] and having misconceptions about NRT use, such as NRT being addictive [31,33,35,36]. Prior research also highlights to a cultural emphasis on “voluntad propia” or the willpower to quit smoking that is unique to the Hispanic population [36]. In fact, when compared to non-Hispanic adults, Hispanic adults who smoke and have tried to quit smoking are twice as likely to cite willpower as their main reason for not trying pharmacotherapy to aid their quit attempts [19]*.*

### Objectives

To address existing knowledge gaps and build on prior studies [24,25], this secondary analysis used data from an RCT with long-term smoking cessation outcomes comparing 2 smoking cessation apps, iCanQuit (based on ACT) and QuitGuide (following USCPG) [25]. We explored the association between pharmacotherapy use and smoking cessation outcomes in the subsample of 173 English-speaking Hispanic adults recruited nationwide from 30 US states. Given the randomized design, we first tested the potential interaction of pharmacotherapy use and intervention arm on 12-month cigarette smoking abstinence. We then examined whether the use of any pharmacotherapy (ie, NRT, varenicline, or bupropion) augmented each app-based intervention efficacy to help Hispanic adults quit smoking. Finally, we compared NRT (eg, patch, gum, lozenge, and inhaler) users with nonusers, noting the scalability potential of mailed NRT for remote interventions compared to other smoking cessation medications.

## Methods

### Overview

The details of the iCanQuit RCT have been previously published [25]. Adults aged 18 years and older who reported smoking cigarettes every day, who had access to their own smartphones, and who had a desire to quit cigarette smoking within 30 days were eligible to participate. Exclusion criteria were an inability to read English, ongoing smoking cessation treatment including the use of pharmacotherapy at the time of enrollment only, prior use of iCanQuit or QuitGuide app–based interventions, or having a household member already enrolled in the trial. Participants were not excluded from the study if they chose to use pharmacotherapy to aid cessation on their own after randomization.

### Ethical Considerations

All participants provided informed consent. Study procedures were approved by the Fred Hutchinson Cancer Center Institutional Review Board (IRB FHIRB0008317). Participants were compensated up to US $105 for completing data collection at the 3-, 6-, and 12-month follow-ups (US $35 per participant per follow-up). The data is de-identified.

### Recruitment, Study Population, and Enrollment

Recruitment spanned from May 2017 to September 2018. Eligible participants were randomly assigned to iCanQuit or QuitGuide for 12 months. Follow-up data collection spanned from August 2017 to December 2019 via web-based surveys at the 3-, 6-, and 12-month follow-up after randomization. From the original 2415 adults in the iCanQuit parent RCT, a total of 173 Hispanic or Latino participants (173/2415, 7.2% of the total sample) who provided pharmacotherapy data at the 3-month follow-up were included in this paper’s secondary analysis (ie, pharmacotherapy data at the 3-month follow-up were missing for 37 Hispanic participants of 210 enrolled in the study).

### Smartphone App–Based Interventions

The iCanQuit app, described in great detail elsewhere [25], comprises 8 ACT-based levels emphasizing two core processes of ACT: (1) acceptance of cravings to smoke, including physical sensations, emotions, and thoughts that cue smoking, and (2) aligning personal values, such as family, community, and health, with a smoke-free life. The “preparing to quit” phase fosters acceptance through mindfulness. It includes an “urge help” function and encourages users to track urges and cigarettes smoked daily. The “after you quit” phase focuses on motivation and relapse prevention.

QuitGuide, a free app developed by the NCI that is based on the USCPGs for smoking cessation [28], offers educational resources and techniques for quitting smoking and preventing relapse. It provides information on smoking cravings and quitting barriers. Unlike iCanQuit, which motivates through personal life values, QuitGuide focuses on health consequences and avoidance of cravings to smoke. Neither the content of iCanQuit nor QuitGuide was specifically designed for Hispanic adults who smoke. Neither arm provided pharmacotherapy to aid smoking cessation, coaching, or other interventions. Both apps educated participants on FDA-approved medications to aid smoking cessation, highlighting benefits, where to obtain them, use instructions, and common side effects.

### Measures

#### Baseline Characteristics

Participants completed baseline web-based surveys reporting sociodemographic, geographic, and smoking characteristics. Data collected included participants’ self-reported age, sex, race, ethnicity, higher level of education attained, household annual income, employment status, marital status, sexual orientation, and household zip code [37-41]. Baseline smoking characteristics included daily cigarette consumption, cigarette dependence using the Fagerström Test for Cigarette Dependence [42], use of other nicotine-containing tobacco products including e-cigarettes, previous quit attempts, self-confidence in quitting, and friends and family who smoke. Alcohol consumption was assessed using the Quick Drinking Screen [43].

#### Pharmacotherapy Use

At the 3-month follow-up survey, participants were asked if they used NRTs or pharmacotherapy to aid smoking cessation since joining the study (ie, within the first 3 months following randomization). This time frame was selected because the content of each app is designed to be completed within approximately 6 weeks of initial use. Thus, assessing pharmacotherapy use by 3 months provides a relevant timeline to examine its potential synergistic effects with the content of the app-based interventions. Participants who reported using nicotine gum, patch, lozenge, or inhaler and varenicline or bupropion were classified as using pharmacotherapy. Participants selecting gum, patch, lozenge, or inhaler but not varenicline or bupropion were classified as using only NRT.

#### Smoking Abstinence

To assess cigarette smoking abstinence, participants were asked: “When was the last time you smoked, or even tried, a cigarette?” in the 12-month follow-up survey. Response choices were “earlier today,” “24 hours ago,” “2-7 days ago,” “8-30 days ago,” and “over 30 days ago” [25,44]. To be categorized as abstinence for the 30-day primary cessation outcomes, participants needed to report no cigarette consumption in the past 30 days at the time of the 12-month follow-up survey. The primary outcome was 30-day cigarette smoking abstinence analyzed through complete-case analysis and sensitivity analyses including missing-as-smoking and multiple imputation. Additional cessation outcomes included cessation of all nicotine and tobacco products (ie, cigarettes, e-cigarettes or vaping, chewing tobacco, snus, hookahs, cigars, cigarillos, tobacco pipes, and kreteks) and prolonged abstinence from cigarettes.

### Statistical Analysis

Baseline characteristics were compared between groups by use of pharmacotherapy using Fisher exact tests for categorical variables and 2-sided *t* tests for continuous variables. To determine whether pharmacotherapy use or use of NRT alone by 3 months differentially impacted the effect of the app-based intervention arm on the primary 12-month cessation outcome, we used logistic regression models with a pharmacotherapy use or NRT use by intervention interaction term. Logistic regression models were then used to estimate the effect of pharmacotherapy use or NRT use on 12-month cessation, separately for each intervention arm [44]. For multiple imputation of missing 12-month smoking status, we used multivariate imputation by chained equations in the R package *mice* [45]. All models were adjusted for randomization stratification factors from the parent RCT: smoking frequency, education, and depression. Statistical analyses were carried out using R software (version 4.2.3; R Foundation for Statistical Computing), with 2-sided tests and α=.05.

## Results

### Baseline Characteristics

Hispanic participants were recruited from 30 US states, averaging 34.5 years in age, with 50.9% (88/173) being female; 20.2% (35/173) identifying as sexual or gender minority; and 30.6% (53/173) being married or partnered. Regarding race, 59% (102/173) identified as White, 15% (26/173) identified as multiracial, and 13.9% (24/173) did not report their race (Table 1). Regarding education, employment, and income, 46.8% (81/173) had a high school diploma or lower; 45.7% (79/173) were unemployed, disabled, or out of the labor force; and 42.8% (35/173) had annual household incomes of US $20,000 or less. More than half (94/173, 54.7%) screened positive for depression symptoms, 29.8% (51/173) for generalized anxiety, 31.8% (54/173) for panic disorder, 48.2% (82/173) for posttraumatic stress disorder, and 34.5% (59/173) for social anxiety disorder. Additionally, 19.1% (33/173) reported bipolar disorder or schizophrenia. On average, participants reported smoking an average of 16.2 (SD 15.9) cigarettes per day, 46.8% (81/173) had high cigarette dependence, 77.5% (134/173) smoked for at least 10 years, and 24.3% (42/173) were dual users of combustible cigarettes and e-cigarettes.

**Table 1:** Baseline characteristics of Hispanic participants.

Characteristic			Participants, n		Overall (N=173)		Did not use pharmacotherapy (n=135, 78%)		Used pharmacotherapy (n=38, 22%)		*P* value
**Age (years), mean (SD)**			173		34.5 (9.3)		34.2 (9.5)		35.5 (8.5)		.44
**Sex, n (%)**			173								.24
	Female			88 (50.9)		68 (50.4)		20 (52.6)			
	Male			82 (47.4)		65 (48.1)		17 (44.7)			
	Transgender female			1 (0.6)		0 (0)		1 (2.6)			
	Transgender male			2 (1.2)		2 (1.5)		0 (0)			
**Race, n (%)**			173								.19
	American Indian or Alaska Native			8 (4.6)		8 (5.9)		0 (0)			
	Black or African American			13 (7.5)		8 (5.9)		5 (13.2)			
	Multiracial			26 (15)		21 (15.6)		5 (13.2)			
	White			102 (59)		77 (57)		25 (65.8)			
	Unknown (unreported) race			24 (13.9)		21 (15.6)		3 (7.9)			
**LGBT^a^, n (%)**			173		35 (20.2)		28 (20.7)		7 (18.4)		.93
**Education, n (%)**			173								.93
	GED^b^, high school or less education			81 (46.8)		63 (46.7)		18 (47.4)			
	Some college, no degree			63 (36.4)		50 (37)		13 (34.2)			
	College degree or higher			29 (16.8)		22 (16.3)		7 (18.4)			
**Employment status, n (%)**			173								.04
	Employed			94 (54.3)		68 (50.4)		26 (68.4)			
	Unemployed			28 (16.2)		27 (20)		1 (2.6)			
	Disabled			25 (14.5)		18 (13.3)		7 (18.4)			
	Out of labor force			26 (15)		22 (16.3)		4 (10.5)			
**Income (US $), n (%)**			173								.17
	Less than $20,000 per year			74 (42.8)		62 (45.9)		12 (31.6)			
	$20,000-$54,999 per year			77 (44.5)		55 (40.7)		22 (57.9)			
	$55,000 per year or more			22 (12.7)		18 (13.3)		4 (10.5)			
**Married, n (%)**			173		53 (30.6)		43 (31.9)		10 (26.3)		.65
**Rural residence^c^, n (%)**			173		21 (12.1)		19 (14.1)		2 (5.3)		.23
**Mental health positive screening, n (%)**											
	Depression (CES-D)^d^	172		94 (54.7)		70 (52.2)		24 (63.2)		.31	
	General anxiety (GAD-7)^e^	171		51 (29.8)		36 (27.1)		15 (39.5)		.20	
	Panic disorder (ANSQ)^f^	170		54 (31.8)		41 (31.1)		13 (34.2)		.87	
	PTSD (PCL-6)^g^	170		82 (48.2)		60 (45.5)		22 (57.9)		.24	
	Social anxiety (mini-SPIN)^h^	171		59 (34.5)		40 (30.1)		19 (50)		.04	
**Self-reported bipolar or schizophrenia^i^, n (%)**			173		33 (19.1)		26 (19.3)		7 (18.4)		>.99
**Alcohol use, n (%)**											
	Drinks per drinking day, mean (SD)	166		2.3 (4.0)		2.3 (4.2)		2.4 (3.3)		.90	
	Heavy drinking^j^, n (%)	166		29 (17.5)		22 (16.9)		7 (19.4)		.92	
**Smoking behavior**											
	Cigarettes smoked per day, mean (SD)	173		16.2 (15.9)		15.9 (16.2)		17.2 (15.1)		.67	
	FTCD score^k^, mean (SD)	173		5.2 (2.0)		5.3 (1.9)		5.2 (2.5)		.97	
	High nicotine dependence (FTCD≥6), n (%)	173		81 (46.8)		60 (44.4)		21 (55.3)		.32	
	First cigarette within 5 minutes of waking, n (%)	173		75 (43.4)		58 (43)		17 (44.7)		.99	
	Smokes greater than one-half pack (10 cigarettes) per day, n (%)	173		97 (56.1)		72 (53.3)		25 (65.8)		.24	
	Smokes more than 1 pack (20 cigarettes) per day, n (%)	173		18 (10.4)		13 (9.6)		5 (13.2)		.74	
	Smoking for 10 or more years, n (%)	173		134 (77.5)		100 (74.1)		34 (89.5)		.07	
	Used e-cigarettes in the past month, n (%)	173		42 (24.3)		35 (25.9)		7 (18.4)		.46	
	Quit attempts in the past 12 months, mean (SD)	166		3.3 (15.8)		3.8 (17.8)		1.8 (3.2)		.51	
	Confidence to quit smoking^l^, mean (SD)	173		67.9 (28.1)		68.0 (27.6)		67.8 (29.9)		.98	
**Friend and partner smoking**											
	Close friends who smoke, mean (SD)	173		2.8 (1.7)		2.7 (1.7)		2.9 (1.8)		.48	
	Housemates who smoke, mean (SD)	173		1.5 (1.1)		1.5 (1.1)		1.5 (0.8)		.96	
	Living with a partner who smokes, n (%)	173		56 (32.4)		44 (32.6)		12 (31.6)		>.99	

^a^LGBT: lesbian, gay, bisexual, or transgender.

^b^GED: general education development.

^c^To ascertain the residential classification of participants into urban or rural areas within the United States, we used the *zipcode* R library [46] to link participants’ zip codes to geographic locations. The classification was based on rural-urban commuting area codes at the subcounty level [37-41].

^d^Positive screening results for depression via Center for Epidemiologic Studies Depression Scale (CES-D)-20; threshold ≥16 [47].

^e^Positive screening results for generalized anxiety via General Anxiety Disorder (GAD)-7; threshold ≥10 [48].

^f^Positive screening results for panic disorder via Autonomic Nervous System Questionnaire (ANSQ) [49]. A positive screening was recorded for individuals reporting at least 1 panic attack in the past month, provided that at least 1 of these incidents occurred in a situation in which they were not in danger or the center of attention.

^g^Positive screening results for posttraumatic stress disorder (PTSD) via Post-Traumatic Checklist (PCL-6); threshold ≥14 [50].

^h^Positive screening results for social anxiety via Mini-Social Phobia Inventory (mini-SPIN); threshold ≥6 [51].

^i^Presence of bipolar disorder or schizophrenia was self-reported via question: “Do you have any of the following mental health disorders?” Multiselect response options included anxiety disorder, depression disorder, bipolar disorder, schizophrenia, alcohol abuse disorder, drug abuse disorder, or none of the above [52,53].

^j^Heavy drinking is defined as 4 or more drinks on a typical drinking day for women and 5 or more drinks on a typical drinking day for men within the past 30 days [43].

^k^Fagerström Test for Cigarette Dependence (FTCD) score [42].

^l^Range 0-100, where 0 indicates not at all confident and 100 indicates extremely confident.

### Pharmacotherapy Use and Outcome Retention Rates

At the 3-month follow-up, 22% (38/173) of participants used pharmacotherapy (NRT, varenicline, or bupropion). There was no statistically significant difference in pharmacotherapy use between treatment arms (iCanQuit: 16/82, 19.5% vs QuitGuide: 22/91, 24.2%; *P*=.41). Approximately 73.6% (28/38) of participants who reported pharmacotherapy use opted for NRT only (ie, nicotine patch, gum, inhaler, or lozenge), whereas 26.3% (10/38) reported using a combination of NRT and medication (varenicline or bupropion). Outcome data retention at 12 months was high, with an overall rate of 93.6% (162/173). This rate did not differ by intervention arms (iCanQuit: 74/82, 90.2% vs QuitGuide: 88/91, 96.7%; *P*=.09) and pharmacotherapy use (users: 36/38, 94.7% vs nonusers: 126/135, 93.3%; *P*=.82).

### Smoking Abstinence

Report of any FDA-approved pharmacotherapy use by 3 months had a marginal differential impact on 12-month quit rates by intervention arm (*P* for interaction=.053). In the iCanQuit arm, there was a signal indicating higher odds of quitting with pharmacotherapy. Specifically, 12-month cigarette smoking abstinence rates in the iCanQuit arm were 43.8% (7/16) for pharmacotherapy users versus 28.8% (19/66) for nonusers (OR 2.21, 95% CI 0.66-7.48; *P*=.20). In contrast, cigarette smoking abstinence rates in the QuitGuide arm were 9.1% (2/22) for pharmacotherapy users versus 21.7% (15/69) for nonusers (OR 0.36, 95% CI 0.07-1.72; *P*=.20). Similar trends were observed for other secondary smoking cessation outcomes, but none reached statistical significance (Table 2).

The impact of using pharmacotherapy to aid cessation at 12 months was more pronounced when NRT was the sole form of pharmacotherapy used, although the interaction effect between NRT use and app-based intervention arm on the 12-month smoking cessation outcome was not statistically significant (*P*=.09). In the iCanQuit arm, 30-day cigarette smoking abstinence rates at 12 months were 45.5% (5/11) for NRT users versus 28.8% (20/69) for nonusers (OR 2.24, 95% CI 0.54-9.27; *P*=.26). In contrast, in the QuitGuide arm, 30-day cigarette smoking abstinence rates at 12 months were 11.8% (2/17) for NRT users versus 21.7% (15/69) for nonusers (OR 0.49, 95% CI 0.10-2.45; *P*=.36; Table 3).

**Table 2:** Association between any pharmacotherapy use and cessation outcomes in the iCanQuit and QuitGuide treatment arms among Hispanic participants.

12-Month cessation outcome	Interaction between treatment arm and use of pharmacotherapy		Treatment arm	Participants, n	Overall, n (%)	Did not use pharmacotherapy, n (%)	Used pharmacotherapy, n (%)	OR^a^ (95% CI)^b^	*P* value
	β (SE)	*P* value							
30-Day PPA^c^ from cigarettes, complete case	1.97 (1.02)	.053	iCanQuit	74	26 (35.1)	19 (31.7)	7 (50)	2.59 (0.69-9.73)	.16
30-Day PPA from cigarettes, complete case	1.97 (1.02)	.053	QuitGuide	88	17 (19.3)	15 (22.7)	2 (9.1)	0.34 (0.07-1.64)	.18
30-Day PPA from cigarettes, multiple imputation	1.86 (1.02)	.07	iCanQuit	820	290 (35.4)	214 (32.4)	76 (47.5)	2.27 (0.61-8.41)	.23
30-Day PPA from cigarettes, multiple imputation	1.86 (1.02)	.07	QuitGuide	910	177 (19.5)	157 (22.8)	20 (9.1)	0.34 (0.07-1.64)	.18
30-Day PPA from cigarettes, missing-as-smoking	1.79 (0.99)	.07	iCanQuit	82	26 (31.7)	19 (28.8)	7 (43.8)	2.21 (0.66-7.48)	.20
30-Day PPA from cigarettes, missing-as-smoking	1.79 (0.99)	.07	QuitGuide	91	17 (18.7)	15 (21.7)	2 (9.1)	0.36 (0.07-1.72)	.20
30-Day PPA from all nicotine and tobacco products^d^	1.90 (1.03)	.06	iCanQuit	74	21 (28.4)	15 (25)	6 (42.9)	2.79 (0.72-10.77)	.14
30-Day PPA from all nicotine and tobacco products^d^	1.90 (1.03)	.06	QuitGuide	88	16 (18.2)	14 (21.2)	2 (9.1)	0.37 (0.08-1.81)	.22
Prolonged abstinence from cigarettes^e^	1.06 (1.47)	.64	iCanQuit	54	10 (18.5)	8 (17.4)	2 (25)	1.67 (0.27-10.36)	.58
Prolonged abstinence from cigarettes^e^	1.06 (1.47)	.64	QuitGuide	69	6 (8.7)	5 (9.6)	1 (5.9)	0.58 (0.06-5.49)	.64

^a^OR: odds ratio.

^b^OR for use versus no use of pharmacotherapy by 3 months after randomization. All models were adjusted for factors used in stratified randomization: smoking >20 cigarettes per day, high school or lower education, and positive screening for depression.

^c^PPA: point prevalence abstinence.

^d^Including any kind of e-cigarettes or vaping, chewing tobacco, snus, hookahs, cigars, cigarillos, tobacco pipes, and kreteks.

^e^Prolonged abstinence is defined as no smoking since 3 months after randomization, using the self-reported date of the last cigarette.

**Table 3:** Association between use of nicotine replacement therapy (NRT) only and cessation outcomes in the iCanQuit and QuitGuide treatment arms among Hispanic participants.

12-Month cessation outcome	Interaction between treatment arm and use of NRT,		Treatment arm	Participants, n	Overall, n (%)	Did not use NRT, n (%)	Used NRT, n (%)	OR^a^ (95% CI)^b^	*P* value
	β (SE)	*P* value							
30-Day PPA^c^ from cigarettes, complete case	1.88 (1.11)	.09	iCanQuit	69	24 (34.8)	19 (31.7)	5 (55.6)	3.09 (0.62-15.41)	.17
30-Day PPA from cigarettes, complete case	1.88 (1.11)	.09	QuitGuide	83	17 (20.5)	15 (22.7)	2 (11.8)	0.47 (0.09-2.33)	.36
30-Day PPA from cigarettes, multiple imputation	1.69 (1.11)	.13	iCanQuit	770	270 (35.1)	214 (32.4)	56 (50.9)	2.46 (0.51-11.90)	.27
30-Day PPA from cigarettes, multiple imputation	1.69 (1.11)	.13	QuitGuide	860	177 (20.6)	157 (22.8)	20 (11.8)	0.47 (0.10-2.35)	.36
30-Day PPA from cigarettes, missing-as-smoking	1.56 (1.06)	.14	iCanQuit	77	24 (31.2)	19 (28.8)	5 (45.5)	2.24 (0.54-9.27)	.26
30-Day PPA from cigarettes, missing-as-smoking	1.56 (1.06)	.14	QuitGuide	86	17 (19.8)	15 (21.7)	2 (11.8)	0.49 (0.10-2.45)	.39
30-Day PPA from all nicotine and tobacco products^d^	1.66 (1.11)	.14	iCanQuit	69	19 (27.5)	15 (25)	4 (44.4)	2.73 (0.54-13.7)	.22
30-Day PPA from all nicotine and tobacco products^d^	1.66 (1.11)	.14	QuitGuide	83	16 (19.3)	14 (21.2)	2 (11.8)	0.51 (0.10-2.54)	.41
Prolonged abstinence from cigarettes^e^	0.29 (1.68)	.91	iCanQuit	51	9 (17.6)	8 (17.4)	1 (20)	1.20 (0.11-12.87)	.88
Prolonged abstinence from cigarettes^e^	0.29 (1.68)	.91	QuitGuide	64	6 (9.4)	5 (9.6)	1 (8.3)	0.90 (0.09-9.10)	.93

^a^OR: odds ratio.

^b^OR for use versus no use of NRT by 3 months after randomization. All models were adjusted for factors used in stratified randomization: smoking >20 cigarettes per day, high school or lower education, and positive screening for depression.

^c^PPA: point prevalence abstinence.

^d^Including any kind of e-cigarettes or vaping, chewing tobacco, snus, hookahs, cigars, cigarillos, tobacco pipes, and kreteks.

^e^Prolonged abstinence is defined as no smoking since 3 months after randomization, using the self-reported date of the last cigarette.

## Discussion

### Principal Findings

This paper aimed to address the critical knowledge gaps regarding the efficacy of smoking cessation app–based interventions combined with pharmacotherapy to help individuals quit cigarette smoking, particularly among Hispanic adults who tends to underuse evidence-based smoking cessation treatments, including pharmacotherapy [17,25,26,54]. The findings suggest that self-selected use of pharmacotherapy, including NRT, varenicline, or bupropion, improved 12-month quit rates among participants in the iCanQuit arm (users: 7/14, 50% vs nonusers: 19/60, 31.7%). However, this effect was not observed among participants in the QuitGuide arm (users: 2/22, 9.1% vs nonusers: 15/66, 22.7%).

The differential effect of using pharmacotherapy between participants in the iCanQuit and QuitGuide arms suggests that combining pharmacotherapy with an ACT-based intervention (iCanQuit) may have synergistically helped participants manage smoking cravings during quit attempts. The results suggest a combined and synergetic effect of managing short- and long-term withdrawal symptoms, resulting in a more effective treatment than ACT-based behavioral interventions alone. It is plausible that short-term withdrawal symptoms were ameliorated using pharmacotherapy, while the skills using iCanQuit to manage withdrawal symptoms may have helped in the long term. While the QuitGuide app–based intervention also mentions FDA-approved pharmacotherapy to aid cessation, the app usually portrays medications as separate rather than complementary to learning behavioral skills to quit smoking [25,28]. It is then possible that participants in the QuitGuide arm may have not understood the potential complementary role of using pharmacotherapy to aid smoking cessation alongside the app. It is plausible that guidance is needed when pharmacotherapy is offered alongside behavioral interventions to have the desired synergetic effect [28]. Further investigation is warranted to understand the improved quit rates with pharmacotherapy in iCanQuit but not QuitGuide.

It is important to note that Hispanic adults in this study opted independently to use pharmacotherapy to aid smoking cessation, albeit at a nonsignificantly lower rate than non-Hispanic participants (38/173, 22% vs 581/1915, 30.3%; *P*=.33). This is contrary to population-based reports among US Hispanic adults being 50% less likely to use evidence-based smoking cessation treatments compared to the general population of adults who smoke in the United States [17,26,54]. Our results showed the potential of evidence-based smoking cessation interventions that are delivered remotely in improving awareness of FDA-approved pharmacotherapy to aid cessation.

### Limitations

This study has several limitations. First, the reliance on secondary data inherently limits findings, as participants were not randomized to receive pharmacotherapy, resulting in associations rather than causal effects. Second, details on dosage, adherence, and duration of pharmacotherapy use are unknown; thus, the nature of pharmacotherapy usage remains uncertain. Third, Hispanic participants were English speakers who smoked daily, which limits the generalizability of the findings to Spanish-speaking Hispanic adults, or those who smoke less than daily [55]. Finally, reliance on self-reported use of pharmacotherapy and smoking abstinence introduces subjectivity and reporting bias. Future multimethod assessment protocols for smoking behavior would therefore be important.

This study also had several strengths. First, it is the first study to explore the effect of an app-based smoking cessation intervention combined with pharmacotherapy use as a potential treatment for Hispanic adults who smoke and wish to quit [17,26,54]. Second, the trial’s long-term smoking cessation outcomes (12 months) and high retention rates that did not differ by intervention arm or pharmacotherapy use helped reduce any potential bias in the complete-case analysis and missing-as-smoking sensitivity analyses. Third, despite the modest sample size, Hispanic participants were recruited from 30 US states, underscoring the geographic diversity of the results and the potential of app-based interventions for widespread reach and broad dissemination.

The results from this secondary analysis have significant public health implications for designing future interventions to help Hispanic adults quit smoking. First, app-based smoking cessation interventions offer a viable approach for hard-to-reach population who are lacking access to evidence-based treatments, including 24% of uninsured Hispanic adults in the United States [32]. Second, app-based delivered interventions could help overcome accessibility barriers like transportation and time constraints. Third, our results show a notable increase in quit rates when using NRT alongside iCanQuit compared to nonuse (5/9, 55.6% vs 20/63, 31.7%). This is noteworthy, as over-the-counter NRT has shown high reach and efficacy [16,29]. Additionally, NRT is readily available and cost-effective and requires minimal medical oversight compared to other medications (varenicline or bupropion) [56,57]. Mailed NRT [58,59], as seen in previous trials, could hold potential for broad dissemination and scalability of smoking cessation programs at the population level. Moreover, future interventions for the Hispanic population could be offered in Spanish, expanding their reach to the 28% of Spanish-speaking Hispanic adults currently living in the United States [32].

### Conclusions

This secondary analysis underscores the nuanced role of pharmacotherapy in enhancing the efficacy of app-based smoking cessation interventions among Hispanic adults. Pharmacotherapy use, particularly when paired with an ACT-based app (iCanQuit), appears to improve quit rates, highlighting a potential pathway to address disparities in smoking cessation outcomes. In contrast, the USCPG-based app (QuitGuide) did not result in a similar synergistic effect when combined with pharmacotherapy. Considering the scalability of NRT, future research should evaluate the efficacy and cost-effectiveness of remotely providing NRT alongside iCanQuit compared to iCanQuit alone.

## Appendix

Multimedia Appendix 1 CONSORT-eHEALTH checklist (V 1.6.1).

## Abbreviations

ACT: acceptance and commitment therapy
FDA: Food and Drug Administration
NCI: National Cancer Institute
NRT: nicotine replacement therapy
OR: odds ratio
RCT: randomized controlled trial
USCPG: US clinical practice guideline

## Data Availability

The datasets generated and analyzed during this study are available from the corresponding author on reasonable request.
